# Pop Culture in the Classroom: Associations with Student Learning Outcomes and the Underlying Psychological Mechanisms

**DOI:** 10.3390/bs15060731

**Published:** 2025-05-24

**Authors:** Su Tao, Yuchen Yang

**Affiliations:** Institute of Ideological and Political Education Psychology, School of Marxism, China University of Geosciences (Beijing), Beijing 100083, China; yangyuchen@email.cugb.edu.cn

**Keywords:** pop culture pedagogy, learning outcomes, learning satisfaction, academic performance, learning engagement

## Abstract

As an emerging pedagogical strategy, the integration of pop culture into university classrooms has gained increasing attention. This study employs a quasi-experimental design to investigate the potential benefits and psychological mechanisms of incorporating pop culture into higher education curricula. Data were collected from 511 students across two courses and four class sections through both survey responses and in-class essay evaluations. The results indicate that students reported higher learning satisfaction with courses incorporating pop culture compared to traditional courses. However, no significant differences were observed in essay scores between the two groups. Mediation analysis revealed that the association between pop culture integration and increased satisfaction was primarily driven by student learning engagement and the perceived functionality of the case materials. Notably, for students who had not previously been exposed to the pop culture references used in class, the pattern was reversed, suggesting a potential risk of “Pop Culture Alienation”. These findings provide empirical evidence for the pedagogical value of pop culture in higher education while underscoring the need to address its differential impact on diverse student populations.

## 1. Introduction

Popular culture refers to the widely disseminated, accepted, and participated cultural expressions and practices that are characteristic of a particular society at a specific historical moment, often serving as shared forms of identity and everyday experience (Delaney, 2007; Fedorak, 2018). Examples of pop culture span a variety of genres, including film and television, popular novels, pop music, social media, and internet slang. Compared with traditional cultural forms, pop culture is distinguished by its accessibility, diffusibility, entertainment value, and temporality, making it particularly influential and well-received among young learners (Singh, 2022). It has even been viewed as a medium through which individuals construct personal and social identities (Literat & Kligler-Vilenchik, 2021; Yu et al., 2025).

As a contemporary pedagogical strategy, pop culture pedagogy has increasingly been incorporated into university classrooms across various disciplines such as psychology, economics, media studies, and linguistics (Collins, 2012; Duff, 2003; Mahiri, 2000; Maudlin & Sandlin, 2015; Peacock et al., 2018; Wooten et al., 2021). However, most of the current literature consists of theoretical or qualitative research, and several key issues surrounding pop culture pedagogy remain unresolved. For instance: Can the learning outcomes of integrating pop culture into higher education be quantitatively assessed? Are the learning outcomes mediated by specific psychological mechanisms? To what extent do individual differences (e.g., cultural background) influence the perceived value or impact of this pedagogical approach—i.e., are there identifiable boundary conditions? These questions call for further theoretical clarification and rigorous empirical investigation.

### 1.1. Effectiveness of Integrating Pop Culture into University Classrooms

The use of pop culture in education has emerged as a prominent topic of scholarly interest, with numerous studies examining its influence on learning outcomes. Existing research demonstrates that integrating elements of pop culture into university classrooms not only enhances students’ emotional engagement (Dietrich et al., 2021) but also significantly improves their critical thinking skills (Jubas, 2023). Incorporating pop culture texts into academic curricula was also shown to enrich professional education and foster a stronger sense of disciplinary identity (Jubas et al., 2025). Moreover, pop culture contributes to the development of students’ communicative competence (Kostadinovska-Stojchevska et al., 2015) and leadership abilities (Devies et al., 2025). Its interdisciplinary and multicultural nature supports the cultivation of multi-perspectivity and the integration of knowledge across disciplines (Balogun & Aruoture, 2024; Qian et al., 2023).

In educational practice, the integration of pop culture as a pedagogical tool has been especially prominent in the fields of humanities and social sciences. Within these disciplines, pop culture texts are often utilized to guide students in the critical exploration of human behavioral patterns, group dynamics, and large-scale social phenomena such as gender, class, and race (Taber et al., 2017). For example, through the analysis of popular films, educators can help students understand theoretical concepts such as gender stereotypes and identity construction (Taber, 2015; Taber et al., 2017). The use of K-pop in the classroom can mirror cultural and economic trends occurring beyond the academic context, thereby aiding students in comprehending economic principles (Wooten et al., 2021). In linguistics courses, the analysis of internet slang and memes has also proven to be an effective entry point for exploring language evolution and the interplay between language and sociocultural dynamics (Duff, 2003). These practices suggest that pop culture is not merely a pedagogical aid but also a powerful educational strategy capable of fostering higher-order cognitive skills. Therefore, pop culture can serve as a source of transformative learning (Litawa, 2023).

### 1.2. Psychological Mechanisms Underlying the Pedagogical Impact of Pop Culture

Several studies analyzed the psychological mechanisms through which pop culture enhances learning outcomes in higher education. For instance, Litawa (2023) argued that learning processes involving popular art integrate three key dimensions of learning—cognitive, emotional, and social. The effectiveness of pop culture in promoting learning outcomes lies in its unique cultural attributes, which align closely with the learning characteristics of contemporary college students.

Firstly, as Digital Natives, contemporary college students have an inherent receptivity to pop culture (Prensky, 2001). This cultural affinity may enhance their learning engagement and boost motivation. To attract students’ interest and foster motivation and engagement, the use of pop culture themes has become a transversal trend in higher education (Picault, 2019). Existing research demonstrated that teaching-related factors are significant predictors of student engagement (Li & Xue, 2023). Short videos, for instance, were shown to capture students’ attention in the classroom (Wooten, 2020). When instructors incorporate elements of pop culture—such as films, TV shows, and trending topics—into their teaching, it can stimulate students’ curiosity (Jubas, 2023) and support their engagement and interest (Ben Abdesslem & Picault, 2023; Dietrich et al., 2021). Pop culture pedagogical strategies not only enhance students’ involvement but also encourage them to participate actively in discussions and express their perspectives (Kostadinovska-Stojchevska et al., 2015). Moreover, from a behaviorist standpoint, linking academic material with pop culture—often associated with enjoyment and positive emotions—can increase the overall sense of pleasure in the learning process, thereby reinforcing engagement (Zeng et al., 2023). Both student engagement and the learning emotion of enjoyment were found to play critical roles in promoting student learning outcomes in the higher education context (Li & Xue, 2023; Lüftenegger et al., 2016; Wang et al., 2025).

Secondly, elements of pop culture help construct meaningful connections between academic knowledge and real-life experiences, thereby enhancing students’ perceived course relevance. A stronger sense of perceived task value arises when learners recognize that course materials are substantively related to their everyday lives, which, in turn, leads them to view the knowledge as personally meaningful and developmentally important. For example, instructors may use films to raise questions about pressing social issues (Brown, 2011) or analyze gender representations in popular television series to introduce students to perspectives rooted in social constructionism (Taber, 2015). These approaches make the life relevance of abstract academic concepts more tangible, enhancing both their explanatory power and practical significance. When students perceive greater relevance in course content, their motivation and academic performance are significantly improved (Fedesco et al., 2017; Pérez-Pérez et al., 2020). The situated learning theory (Lave & Wenger, 1991) further emphasizes that knowledge acquisition and application must be embedded within specific cultural practices and social contexts. The simulated social scenarios presented through pop culture enhance the authenticity of learning content and may also provide opportunities for participation in a “community of practice”, facilitating knowledge transfer and real-world application. In doing so, pop culture-based instruction can effectively address the common problem of inert knowledge (Trench & Minervino, 2017) found in traditional educational settings.

Third, the more important instructional effect of pop culture lies in its promotion of meaningful learning. In terms of classroom information processing, the Adaptive Control of Thought–Rational (ACT-R) theory posits that information encountered more frequently attains higher activation levels and is thus retrieved more easily and rapidly (Anderson et al., 1998; Krivec & Guid, 2020). Because pop culture permeates students’ daily lives far more than traditional academic examples—and often carries a more positive valence—it naturally achieves higher activation and facilitates quicker retrieval. Previous research noted that the narrative and affective features inherent to pop culture resonate with students’ lived experiences, thereby enhancing their critical thinking (Brown, 2011; Jubas, 2023; Peacock et al., 2018). Additionally, according to Ausubel’s theory of cognitive assimilation (Ausubel, 1963; Bryce & Blown, 2024), students’ pre-existing cognitive structures can serve as advance organizers, providing cognitive anchors for the assimilation of new knowledge. Pop culture can be conceptualized as a cognitive scaffold that, by constructing comprehensible contexts, enables abstract concepts to be concretized, grasped, and internalized. In sum, we argue that pop culture not only stimulates learning interest but also, at the cognitive level, provides a meaningful context that significantly facilitates information processing and knowledge retention.

### 1.3. Challenges of Integrating Pop Culture into Teaching

While integrating pop culture into university teaching may offer significant benefits—such as enhancing student engagement and supporting knowledge acquisition—its implementation still faces several critical challenges (Duncan-Andrade, 2004). Chief among these are concerns regarding the quality of pop culture materials and the need to uphold instructional equity.

First, the most important predictors of students’ satisfaction were learning content and course design (Barbera et al., 2013) as well as information quality (Pérez-Pérez et al., 2020). As a prominent form of mass culture, pop culture is inherently characterized by entertainment and commercialization. These features stand in subtle tension with the academic rigor and seriousness pursued in higher education (Jubas et al., 2025), leading to concerns about the quality of its content. These challenges manifest on three levels: (1) Difficulties in content selection, particularly due to the widespread tendencies toward fragmentation and superficiality within internet subcultures (Ulusoy & Fırat, 2016). (2) Challenges in ensuring curriculum relevance. Instructors need to avoid the pitfalls of superficial analogies or forced connections between pop culture materials and course content. As noted by Marquis et al. (2020b), “problems finding appropriate materials” is one of the most frequently reported challenges in using film and video in teaching and learning. (3) Challenges in establishing academic legitimacy. A persistent mindset among some students and faculty is the binary opposition between entertainment and academia, in which representations in popular films are often dismissed as “just entertainment” (Marquis et al., 2020a). These call for instructors to possess a high level of cultural literacy, enabling them to identify exemplary cases—those that not only offer intellectual depth but also align closely with instructional objectives.

Secondly, differences in cultural acceptance give rise to concerns regarding educational equity. Youths’ exposure to pop culture varies significantly, leading to diverse individual cultural preferences (Balogun & Aruoture, 2024). This cultural heterogeneity in exposure may lead to a comprehension gap in the classroom. While college students generally display a high level of acceptance toward pop culture, those who are unfamiliar with or disinterested in the specific examples used may struggle to form substantial connections between cultural phenomena and academic concepts. Some may even perceive the cultural elements as mere entertainment tools, thereby questioning their academic value (Marquis et al., 2020a). Furthermore, according to the Shared Reality Theory (Echterhoff & Higgins, 2018), students who have not been previously exposed to pop culture may struggle to establish a shared reality with their peers. This could weaken their interpersonal relationships, leading to less participation in class discussions and even feelings of exclusion. This dynamic may lead to a decrease in learning engagement and satisfaction, ultimately undermining instructional effectiveness.

In summary, this study employs a quasi-experimental design to compare the learning outcomes of university classes utilizing pop culture cases versus traditional ones. Learning outcomes are assessed along two dimensions: students’ subjective perceptions (i.e., self-reported satisfaction) and objective academic performance (i.e., essay scores). The research addresses whether pop culture-integrated instruction is associated with differences in students’ subjective and objective learning outcomes compared to traditional instruction, what psychological mechanisms may mediate these associations, and whether certain student groups, such as those without prior exposure to pop culture materials, might experience less benefit from pop culture integration.

Correspondingly, the following hypotheses were proposed:

**H1.**  *Integrating pop culture into classes will be associated with higher subjective and objective learning outcomes.*

**H2.**  *This association will be mediated by increased student learning engagement, perceived course relevance, and perceived cognitive functionality of the cases. Conversely, perceived case quality may serve as a negative mediator.*

In addition, exploratory analyses were conducted to examine the reactions of students with no prior exposure to pop culture materials, aiming to uncover potential challenges and limitations associated with the practical implementation of pop culture pedagogy.

## 2. Materials and Methods

### 2.1. Participants

The students were all from a comprehensive university in Beijing, China, and were recruited from two university-wide elective courses. The first course, Social Psychology, was an undergraduate elective with 120 students per class. After excluding absent students and those with missing data, the final dataset included 228 students. The second course, Introduction to the Dialectics of Science, was a graduate-level core course with 145 students per class. After excluding absent students and those with missing data, the final valid dataset consisted of 283 students.

Both courses included students from a wide range of academic disciplines across the university. The gender distribution was 286 male and 225 female students, with an average age of 20.85 ± 1.82 years. Participant demographics by course and group are presented in Table 1.

### 2.2. Procedure

A quasi-experimental design was adopted, with intact classes assigned to either the pop culture group or the control group. For each course, two class sections were involved: one assigned to the pop culture group and the other to the control group. No randomization was applied at the individual level; instead, intact classes were assigned to different instructional conditions based on course scheduling. The two sections of the Social Psychology course were conducted over two semesters, whereas the two sections of the Introduction to the Dialectics of Science course were offered in the same semester as parallel sections. All core case presentations and learning outcome assessments were conducted during the first class session of each course to ensure that students had comparable baseline knowledge levels prior to instruction. The same instructor taught both sections of each course to minimize potential differences in teaching style that might affect students’ satisfaction ratings.

The Social Psychology course consisted of three class hours, while Introduction to the Dialectics of Science comprised four class hours. The core case presentation lasted approximately 20 min and was referenced multiple times throughout the course. The assessment of learning outcomes took approximately 30 min. The pop culture group and control group had identical course designs, except for the core case.

#### 2.2.1. Pop Culture Group

In Social Psychology class, the Chinese blockbuster film *NeZha 2* (which, as of 26 March 2025, grossed over $2.1 billion, ranking fifth in global box office history) was used as a case study. Students analyzed the psychological motivations behind audience engagement with the film to illustrate core research areas in social psychology. Meanwhile, the Introduction to the Dialectics of Science course incorporated references from the renowned Chinese science fiction novel *The Three-Body Problem*, which was recently adapted into film and television, sparking widespread discussion. Through analyzing its depictions of artificial intelligence and facial recognition, the course explores the relationship between fundamental theoretical innovation and technological development. The cases were presented using multiple images and text rather than videos, to avoid the additional media effects associated with pop culture materials such as animated films.

#### 2.2.2. Control Group

In the corresponding sessions, traditional teaching cases were used. In the Social Psychology course, students analyzed the motivations behind course selection behavior. In the Introduction to the Dialectics of Science course, they examined the relationship between fundamental theoretical innovations and technological advancements throughout past industrial revolutions. The cases were also presented using multiple images and text, with the organization and duration roughly equivalent to those of the pop culture cases.

At the end of the course, all students were required to complete a 20-min in-class essay of approximately 300 Chinese characters (equivalent to about 180–200 English words) to assess objective learning outcomes, primarily evaluating their knowledge of the course content. The essay topic for the Social Psychology course was “Please analyze the main differences between the research perspectives of social psychology and related disciplines such as sociology”, while the topic for the Introduction to the Dialectics of Science course was “Please analyze the primary factors influencing scientific and technological progress”. There was no specific requirement for students to reference the core case in their essays. Subsequently, students completed a course evaluation survey to assess the subjective learning outcomes and the proposed mediators.

### 2.3. Informed Consent

Students were required to complete the in-class essay to earn course credit, whereas the course evaluation survey was voluntary and non-mandatory.

The purpose and significance of the study were prominently stated at the beginning of the course evaluation survey, which only indicated that it was an assessment of teaching effectiveness, without mentioning pop culture. Students were informed that they could withdraw from the survey at any point during the completion process. Additionally, students were reassured that their responses, whether positive or negative, would not affect their course grades. Oral informed consent was obtained from all students when they submitted the survey.

The survey data were entered by a third party with no conflict of interest with the course instructors. Essay scores were linked to survey responses using student ID numbers, followed by data anonymization procedures to ensure anonymity. The course evaluation results had no impact on students’ final grades.

### 2.4. Measures

#### 2.4.1. Learning Outcomes

Learning outcomes were assessed using both students’ self-reports and objective indicators (Stark et al., 1998). Subjectively, students rated their overall learning satisfaction using a three-item scale: (1) “How satisfied are you with this course?”; (2) “How would you evaluate the instructor’s overall teaching effectiveness?”; and (3) “Would you recommend this course to other students?”. Responses were rated on a five-point Likert scale (1 = very dissatisfied/not recommend at all, 5 = very satisfied/strongly recommend). The scale demonstrated good internal consistency (Cronbach’s α = 0.81). Students’ objective learning outcomes were assessed through their scores on an in-class essay assignment, graded by the course instructor, with a maximum score of 10 points.

#### 2.4.2. Learning Engagement

Based on the conceptualization by Li and Xue (2023), students’ learning engagement in class was measured using four items that assessed their interest and attentiveness during classroom activities: (1) “I find the course content interesting”, (2) “I think the course content is engaging”, (3) “I stay focused while studying this course”, and (4) “I actively participate in class”. Students rated each item on a five-point Likert scale (1 = strongly disagree, 5 = strongly agree), with the scale demonstrating acceptable internal reliability (Cronbach’s α = 0.76).

#### 2.4.3. Perceived Course Relevance

Drawing on Owen’s (2017) conceptualization, the perceived relevance of the course was measured using two items that captured the extent to which students found the course content to be personally meaningful: (1) “The knowledge from this course is very important to me” and (2) “The knowledge from this course is closely related to my studies and daily life”. Ratings were on a five-point Likert scale (1 = strongly disagree, 5 = strongly agree), with an acceptable internal reliability (Cronbach’s α = 0.86).

#### 2.4.4. Case Evaluation

Students also evaluated the core case used in the course across three dimensions. First, based on concerns about the potential quality issues of cases involving pop culture integration into the classroom (Maudlin & Sandlin, 2015), perceived case quality was assessed using three items: (1) “This case is highly relevant to the course content”, (2) “This case demonstrates strong academic value”, and (3) “This case is well-structured and organized” (Cronbach’s α = 0.81). Second, based on the potential cognitive impact of pop culture integration, perceived case functionality was measured using three items: (1) “This case helps me better understand the course content”, (2) “This case encourages deeper thinking about the subject matter”, and (3) “This case aids in my retention of course knowledge” (Cronbach’s α = 0.84). Third, case familiarity was assessed with three items: Students were first asked, “Have you heard of this case before?” (Control Group)/“Have you seen or read this work before?” (Pop Culture Group). They then rated (1) “How familiar are you with this case/work?” and (2) “How much do you like this case/work?” on a five-point Likert scale (1 = not familiar at all/strongly dislike, 5 = very familiar/strongly like).

### 2.5. Data Analysis

Data analysis was conducted using SPSS 26.0 (IBM Corp., Armonk, NY, USA). First, descriptive statistics and correlation analyses were performed on the key variables. Then, independent-samples *t*-tests were used to examine differences in learning outcomes associated with pop culture integration, with mediation analyses conducted using the PROCESS macro Version 4.3.1. Subsequently, ANOVA was employed to explore the moderating role of case exposure in shaping these differences, followed by mediation analyses using PROCESS to investigate the underlying mechanisms.

## 3. Results

### 3.1. Descriptive Statistics and Correlation Analysis

Descriptive statistics and Pearson correlation analyses were conducted for all key variables, as presented in Table 2. Results indicated that learning satisfaction was significantly associated with learning engagement, perceived course relevance, and both perceived case quality and functionality. However, it was not significantly related to the objective measure of learning outcomes (i.e., essay score). Essay score was only weakly correlated with students’ learning engagement (*r* = 0.10, *p* = 0.020) and not significantly associated with other subjective measures.

### 3.2. Group Differences in Learning Outcomes and Proposed Mediators

Independent-samples t-tests were conducted with group (pop culture vs. control) as the independent variable and the learning outcomes and proposed mediators as dependent variables. As shown in Table 3, the results revealed that the pop culture group scored significantly higher than the control group on learning satisfaction, learning engagement, and perceived case functionality. However, no significant differences were found between the two groups in essay scores, perceived course relevance, or perceived case quality.

To examine whether course type was associated with learning outcomes, two separate ANOVAs were conducted with group and course type as independent variables and satisfaction and essay score as dependent variables, respectively. The main effect of course type was not significant for either outcome (for satisfaction, *F* = 0.002, *p* = 0.975; for essay score, *F* = 3.66, *p* = 0.307), nor was the interaction between course type and group (for satisfaction, *F* = 3.36, *p* = 0.067; for essay score, *F* = 0.06, *p* = 0.809). These results suggest that different course types were not significantly linked to differences in learning outcomes in this study.

### 3.3. Mediation Analysis of Pop Culture Integration on Learning Satisfaction

**Figure 1:**
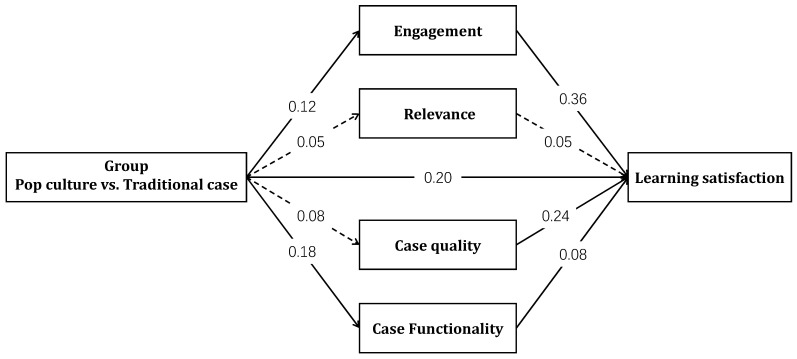
Mediation model of group differences in learning satisfaction. Note: solid lines = significant paths (*p* < 0.05); dashed lines = non-significant paths (*p* > 0.05).

Given the lack of significant association between group assignment (pop culture vs. control) and students’ objective academic performance (i.e., essay scores), the mediation analysis focused solely on subjective learning outcomes—specifically, learning satisfaction. All continuous variables were mean-centered before analysis. The mediation model was tested using Model 4 in PROCESS v4.1 for SPSS, with group assignment as the independent variable; learning engagement, perceived course relevance, perceived case quality, and functionality as parallel mediators; and learning satisfaction as the dependent variable. Demographic variables and course type were included as control variables. The results of the mediation analysis are presented in Table 4 and illustrated in Figure 1.

Bootstrap analysis revealed that the total effect of group assignment on learning satisfaction was significant, *B* = 0.28, *SE* = 0.05, *t* = 5.47, and *p* < 0.001. The indirect effect via engagement was significant (*effect* = 0.04, *BootSE* = 0.02, 95% CI [0.02, 0.08]) as was the effect via case functionality (*effect* = 0.02, *BootSE* = 0.01, 95% CI [0.00, 0.04]). Although perceived case quality was a significant predictor of satisfaction, it was not significantly affected by group assignment and thus did not serve as a mediator (*effect* = 0.02, *BootSE* = 0.02, 95% CI [−0.02, 0.06]). Similarly, course relevance was associated with course type but not with either group or satisfaction, rendering its indirect effect non-significant (*effect* = 0.00, *BootSE* = 0.00, 95% CI [−0.01, 0.01]). The mediating variables jointly accounted for 28.57% of the total effect.

### 3.4. Conditional Effects of Pop Culture Integration

Next, the study explored the impact of exposure to cases used in the course on learning outcomes. Among the 256 students in the pop culture group, 47 students had never been exposed to the work referenced in the class, accounting for 18.4% of the group. In contrast, 21 students in the control group, out of 255, had not heard of the case, representing 8.24% of the group.

**Figure 2:**
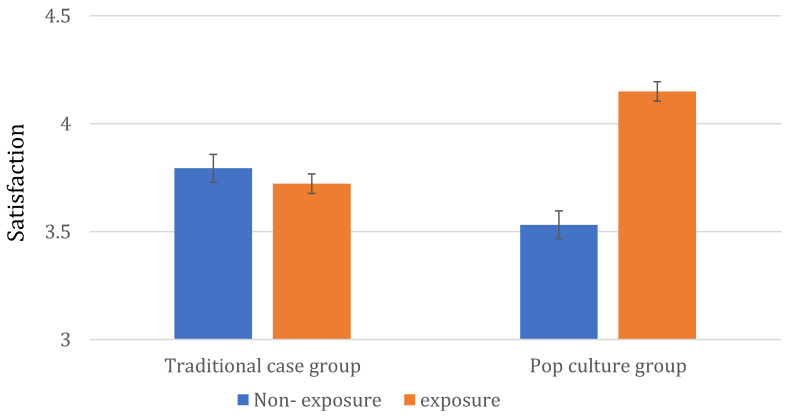
Impact of prior exposure to course-referenced case on learning satisfaction.

To examine how prior exposure to the cases referenced in the course affected learning satisfaction, a two-way analysis of variance (ANOVA) was conducted with group and exposure as independent variables and learning satisfaction as the dependent variable. The results indicated that neither the main effects of group (*F* = 0.06, *p* = 0.850) nor exposure (*F* = 0.63, *p* = 0.573) were significant. However, the interaction effect between group and exposure was significant (*F* = 19.34, *p* < 0.001, *η*^2^ = 0.037). The means for each group are presented in Figure 2.

The simple effects test for the interaction revealed that, in the traditional case group, prior exposure to the case had no significant impact on learning satisfaction (*F* = 0.31, *p* = 0.577). In contrast, within the pop culture group, students who had previously watched the work (*M* = 4.15, *SD* = 0.56, *N* = 209) reported significantly higher learning satisfaction compared to those who had not (*M* = 3.53, *SD* = 0.62, *N* = 47, *F* = 46.49, *p* < 0.001, *η*^2^ = 0.155). Additionally, students in the non-exposure with pop culture group (*M* = 3.53, *SD* = 0.62, *N* = 47) reported lower learning satisfaction compared to students in the non-exposure with traditional case group (*M* = 3.79, *SD* = 0.56, *N* = 21), with this difference representing marginal significance (*F* = 3.15, *p* = 0.076, *η*^2^ = 0.046).

Next, to further analyze the impact of pop culture exposure on learning outcomes and student psychological responses, independent-sample *t*-tests were conducted using data from the pop culture exposure group. The results are shown in Table 5.

Based on the data in Table 3, it is evident that students who had previously watched the selected pop culture work rated their familiarity and enjoyment significantly higher than those who had not seen it. This suggests that the pop culture work chosen for the course was popular among students and garnered positive reception. Furthermore, exposure to the work significantly influenced students’ satisfaction with the course, although it did not have a significant effect on their essay scores.

**Figure 3:**
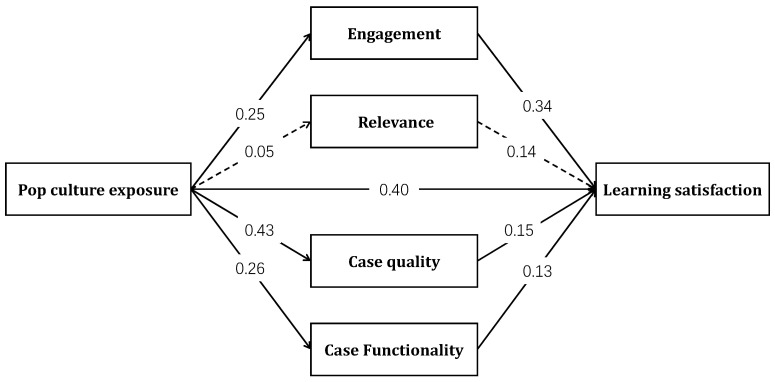
Mediation model of pop culture exposure on learning satisfaction. Note: solid lines = significant paths (*p* < 0.05); dashed lines = non-significant paths (*p* > 0.05).

To explore the mediating process of how pop culture exposure affects learning satisfaction, a mediation analysis was conducted using Process Model 4. The results are presented in Table 6 and Figure 3.

Bootstrap analysis indicated that the total effect of exposure on satisfaction was significant (*total effect* = 0.60, *SE* = 0.09, *t* = 6.51, *p* < 0.001). The mediating effects of learning engagement (*effect* = 0.08, *BootSE* = 0.03, 95% CI [0.03, 0.15]), perceived case quality (*effect* = 0.06, *BootSE* = 0.03, 95% CI [0.01, 0.14]), and case functionality (*effect* = 0.04, *BootSE* = 0.02, 95% CI [0.00, 0.09]) were all significant. However, student-perceived course relevance did not show a significant association with either the independent or dependent variables and did not have a significant mediation effect (*effect* = 0.01, *BootSE* = 0.01, 95% CI [−0.01, 0.04]). The mediating variables jointly accounted for 31.62% of the total effect.

## 4. Discussion

### 4.1. Main Findings of the Study

This study empirically investigated the learning outcomes and psychological mechanisms of incorporating pop culture into university classroom teaching. The findings indicated that, compared to traditional teaching methods, pop culture integration led to higher students’ self-reported satisfaction—a key indicator of subjective learning outcomes—while showing no significant difference in objective learning outcomes, i.e., the quality of student essays. The correlation between learning satisfaction and essay scores was also not significant. These results suggest that the pedagogical value of pop culture may lie more in its ability to influence students’ affective and motivational states rather than directly improving knowledge acquisition. The inconsistency in findings may be attributed to the following three factors. First, objective and subjective learning outcomes are influenced by different factors and do not necessarily converge (Lobanova, 2022; Stark et al., 1998; Tempelaar et al., 2020). Essay quality primarily reflects cognitive learning outcomes, whereas course satisfaction is more closely associated with affective experiences. Second, there may be a time lag in knowledge transformation (Salomon & Perkins, 1989)—interest and motivation sparked by pop culture may require a longer period to translate into measurable cognitive gains. Third, there may be limitations in assessment methods: essay writing may not fully capture other forms of learning benefits potentially associated with the integration of pop culture. These findings provide empirical support for educators seeking to make informed use of pop culture in teaching. They highlight the importance of leveraging its strengths in enhancing the learning experience, while also acknowledging its limitations in promoting deep learning. Such insights can guide the optimal combination of instructional strategies in higher education.

Further mediation analysis revealed that students in the pop culture group reported higher learning satisfaction than those in the traditional instruction group, with this association primarily related to increased students’ classroom engagement and the facilitation of cognitive processes such as understanding, reflection, and memory. Specifically, pop culture elements—due to their inherent appeal and relatability—effectively stimulate students’ interest and engagement, while also promoting meaningful learning by providing concrete and familiar contexts. These two mediation paths were consistent with the research hypotheses. However, the study also found that perceived course relevance and perceived case quality did not mediate the association between case type and learning satisfaction, which did not support the research hypotheses. This may be due to two main reasons. On one hand, course relevance tends to depend more on whether the content is explicitly linked to potential practical or professional applications rather than on specific instructional cases (Owen, 2017). On the other hand, both traditional and pop culture cases can achieve comparable levels of quality when they are well designed and well organized. In this study, no significant difference in perceived quality was found between the pop culture cases and the traditional cases.

Notably, this study introduces the concept of Pop Culture Alienation through an analysis of students’ pop culture exposure. This concept refers to a sense of marginalization experienced by certain students who, due to personal background, sociocultural environment, or economic constraints, have limited exposure to specific forms of pop culture. As a result, they may feel excluded during classroom instruction that incorporates such cultural references. Findings indicate that students in the non-exposure to pop culture group reported even lower learning satisfaction than those in a control group who were unfamiliar with traditional case materials. Further analysis revealed that this group scored significantly lower on several psychological variables, including learning engagement, perceived case quality, and functionality. These variables were found to mediate the relationship between cultural exposure and learning satisfaction. This highlights a potential unintended negative effect of pop culture pedagogy: when students are unfamiliar with the cultural content presented in class, the anticipated positive outcomes may not materialize. Instead, they may experience a sense of alienation that undermines their learning experience and engagement. Overall, this study calls for a more nuanced approach to the integration of pop culture in higher education. While emotional engagement and cognitive development are important goals, equal attention must be paid to cultural inclusivity. Educators should be mindful of diversity and inclusion in course design (Ben Abdesslem & Picault, 2023), ensuring that all students can derive educational value from the materials used, regardless of their cultural exposure.

### 4.2. Theoretical and Practical Implications

First, compared to previous theoretical analyses and qualitative studies, this research provides valuable quantitative empirical evidence supporting the integration of pop culture into university teaching. The findings highlight the crucial role of affective stimulation and motivational maintenance in enhancing students’ learning experiences. This aligns with existing educational theories and empirical results that emphasize the importance of emotional goals in learning (Ben-Eliyahu, 2019; Eynde & Turner, 2006; Krapp, 1999). However, the study also found that incorporating pop culture into teaching was not associated with significant improvements in students’ academic essay performance. This unexpected result offers a new perspective on the cognitive benefits of media and pop culture in education, suggesting that while pop culture can serve as an effective pedagogical tool, its impact on objective learning outcomes may be limited if it fails to promote critical thinking and deeper conceptual understanding.

Second, this study systematically explored the psychological processes through which pop culture relates to learning outcomes, identifying key mediating factors. The results show that pop culture influences learning experiences primarily by increasing students’ learning engagement and facilitating cognitive processes such as knowledge comprehension, thinking, and memory retention. This mechanism provides a theoretical framework for understanding how to effectively incorporate pop culture into classroom practice. It suggests that educators should focus on how to design pop culture cases to optimize students’ cognitive processing, rather than simply introducing such materials for entertainment purposes.

Moreover, the newly proposed concept of Pop Culture Alienation presents an important challenge for inclusive teaching. When course content assumes that all students are familiar with specific forms of pop culture, those with limited exposure to such cultural references may experience a sense of marginalization. This finding underscores the importance of cultural sensitivity in pedagogy. Educators employing pop culture teaching methods must account for the cultural diversity of their students and adopt strategies such as gradual introduction, contextual preparation, and alternative options to ensure that all students can meaningfully participate in the learning process (Kefallinou et al., 2020; Sleeter & Grant, 2008). These strategies are essential not only for fostering equity in the classroom but also for preventing the deepening of cultural disparities in higher education contexts.

### 4.3. Limitations and Future Directions

First, the study design relied on a single course session, which restricts the ability to evaluate the long-term effects. Although pop culture was integrated as a central case study throughout the session, the study’s scope remains limited by the short-term duration. Future research could focus on the long-term impact of integrating pop culture into courses and explore various teaching methods, such as discussions and collaborative learning (Slavin et al., 2003; Terenzini et al., 2001) to further enhance teaching effectiveness.

Second, although baseline control over students’ knowledge levels and satisfaction was attempted by conducting all core case presentations and learning outcome assessments during the first class session and by having the same instructor teach both sections, the absence of a formal pretest remains a limitation of this study. The differences observed between the pop culture and control groups may partially reflect pre-existing differences in participant characteristics, which effects the causal interpretation of the results. Future research could adopt a more rigorous experimental design and incorporate pretest measures to better examine the effects of pop culture integration.

Third, this research was conducted within the context of humanities and social science courses, which naturally align more closely with pop culture. Previous studies showed that instructors in these disciplines are more likely to use pop culture in their teaching and generally hold more favorable attitudes toward it compared to their counterparts in natural sciences and mathematics (Peacock et al., 2018). Although there were attempts to incorporate pop culture into disciplines such as information literacy and chemistry (Behen, 2006; Dietrich et al., 2021), integrating pop culture into STEM courses may face challenges due to a lower degree of relevance. The effects of such integration in STEM fields remain underexplored and warrant further investigation. Future research could examine disciplinary differences in the effectiveness of pop culture integration, especially in fields where its relevance is less immediately evident.

Finally, the phenomenon of pop culture alienation was only explored preliminarily in this study. Students from diverse cultural backgrounds may have varying levels of exposure to, or acceptance of, pop culture, which could influence their engagement with the course content. This issue could be particularly prominent in multicultural settings. Future research should examine how cultural diversity affects the reception of pop culture in the classroom as well as explore the psychological impact on students affected by pop culture alienation. Solutions to address this issue, such as personalized learning approaches or curriculum adjustments (Bernacki et al., 2021; Schmid et al., 2022), should also be considered.

## 5. Conclusions

Integrating pop culture into university classrooms connects academic content with contemporary cultural contexts, thereby promoting comprehension, critical thinking, and memory retention, while also enhancing student learning engagement. As a result, students’ learning satisfaction is significantly improved. However, to achieve optimal academic outcomes, it is crucial to carefully select relevant pop culture materials to strike a balance between entertainment value and academic rigor. Additionally, it is important to consider students who may feel alienated due to limited exposure to pop culture, ensuring that the learning environment remains inclusive and supportive. By achieving this balance, pop culture teaching methods can enrich the overall learning experience and lead to more meaningful academic outcomes.

## Figures and Tables

**Table 1:** Participant demographics and group assignment.

Course	Group	*N*	Age	Sex		Disciplinary Category		
				Male	Female	Humanities and Social Sciences	Natural Sciences	Engineering
Social psychology	Pop culture	112	19.16 ± 0.78	64	48	20	29	71
	Control group	116	19.43 ± 0.76	55	61	33	27	60
Introduction to the dialectics of science	Pop culture	144	21.99 ± 1.43	92	52	0	52	93
	Control group	139	22.21 ± 1.39	75	64	0	64	81

**Table 2:** Descriptive statistics and correlations among key variables (*N* = 511).

	*M ± SD*	Satisfaction	Engagement	Relevance	Quality	Functionality	Essay Score
Satisfaction	3.88 ± 0.61	1					
Engagement	4.05 ± 0.52	0.61 ***	1				
Relevance	4.85 ± 0.43	0.25 ***	0.42 ***	1			
Quality	3.34 ± 0.85	0.60 ***	0.59 ***	0.15 ***	1		
Functionality	3.20 ± 0.85	0.54 ***	0.55 ***	0.14 **	0.79 ***	1	
Essay Score	8.44 ± 0.66	0.08	0.10 *	0.03	0.06	0.01	1

Note: Group: (1, pop culture; 0. control); * *p* < 0.05, ** *p* < 0.01, *** *p* < 0.001.

**Table 3:** Group differences in learning outcomes and proposed mediators.

	Pop Culture (*N* = 256)	Control Group (*N* = 255)	*t*	*p*	*d*
Satisfaction	4.04 ± 0.62	3.73 ± 0.55	5.95	<0.001	0.53
Essay Score	8.46 ± 0.64	8.40 ± 0.67	0.98	0.326	0.09
Engagement	4.12 ± 0.52	3.99 ± 0.51	2.95	0.003	0.25
Relevance	4.88 ± 0.40	4.83 ± 0.46	1.30	0.193	0.12
Quality	3.40 ± 0.87	3.29 ± 0.83	1.44	0.150	0.13
Functionality	3.31 ± 0.86	3.08 ± 0.82	3.09	0.002	0.27

**Table 4:** Mediation analysis of the group differences in learning satisfaction (*N* = 511).

	Engagement (Mediator 1)			Relevance (Mediator 2)			Quality (Mediator 3)			Functionality (Mediator 4)			Satisfaction (Dependent Variable)		
	*B*	*SE*	*t*	*B*	*SE*	*t*	*B*	*SE*	*t*	*B*	*SE*	*t*	*B*	*SE*	*t*
Sex	−0.07	0.05	−1.47	−0.03	0.04	−0.72	−0.14	0.07	−1.91	−0.36	0.07	−5.17 ***	−0.01	0.04	−0.14
Age	−0.02	0.02	−1.24	0.01	0.02	0.70	−0.04	0.03	−1.27	−0.03	0.03	−0.91	−0.04	0.02	−2.69 **
Course type	0.14	0.07	1.96	−0.07	0.06	−1.14	0.52	0.11	4.52 ***	0.56	0.11	5.09 ***	−0.05	0.06	−0.78
Group	0.12	0.05	2.63 **	0.05	0.04	1.30	0.08	0.07	1.06	0.18	0.07	2.55 *	0.20	0.04	5.30 ***
Engagement													0.36	0.05	7.24 ***
Relevance													0.05	0.05	1.05
Quality													0.24	0.04	6.14 ***
Functionality													0.08	0.04	2.15 *
*R* ^2^	0.03			0.01			0.07			0.15			0.51		
*F*	3.83 **			0.87			9.90 ***			22.65 ***			66.56 ***		

Note: Course type: (1, Social Psychology; 2, Introduction to the Dialectics of Science); * *p* < 0.05, ** *p* < 0.01, *** *p* < 0.001.

**Table 5:** Group differences in pop culture exposure on learning outcomes and proposed mediators.

	Exposure (*N* = 209)	Non-Exposure (*N* = 47)	*t*	*p*	*d*
Familiarity	4.44 ± 0.74	1.83 ± 0.76	−21.80	0.000	3.51
Likability	4.28 ± 0.74	2.51 ± 0.72	−14.88	0.000	2.40
Satisfaction	4.15 ± 0.56	3.53 ± 0.62	−6.72	0.000	1.09
Essay Score	8.46 ± 0.66	8.45 ± 0.54	−0.10	0.904	0.02
Engagement	4.17 ± 0.52	3.91 ± 0.46	−3.13	0.002	0.51
Relevance	4.89 ± 0.38	4.83 ± 0.48	−0.89	0.372	0.15
Quality	3.47 ± 0.87	3.09 ± 0.79	−2.78	0.006	0.44
Functionality	3.36 ± 0.88	3.14 ± 0.79	−1.64	0.102	0.25

**Table 6:** Mediation analysis of the effect of pop culture exposure on course satisfaction (*N* = 256).

	Engagement (Mediator 1)			Relevance (Mediator 2)			Quality (Mediator 3)			Functionality (Mediator 4)			Satisfaction (Dependent Variable)		
	*B*	*SE*	*t*	*B*	*SE*	*t*	*B*	*SE*	*t*	*B*	*SE*	*t*	*B*	*SE*	*t*
Sex	−0.11	0.07	−1.65	−0.05	0.05	−0.96	−0.14	0.11	−1.30	−0.38	0.10	−3.70 ***	0.07	0.06	1.13
Age	−0.02	0.03	−1.03	0.00	0.02	0.02	−0.01	0.04	−0.24	−0.02	0.04	−0.37	−0.06	0.02	−2.56 *
Course type	0.09	0.10	0.93	0.00	0.08	−0.05	0.47	0.16	2.87 **	0.52	0.16	3.29 **	−0.02	0.09	−0.21
Exposure	0.25	0.08	2.98 **	0.05	0.07	0.80	0.43	0.14	3.21 **	0.26	0.13	1.97 *	0.41	0.07	5.67 ***
Engagement													0.34	0.07	4.82 ***
Relevance													0.14	0.08	1.77
Quality													0.15	0.05	2.72 **
Functionality													0.13	0.06	2.45 *
*R* ^2^	0.05			0.01			0.10			0.14			0.53		
*F*	3.42 **			0.43			7.01 ***			10.29 ***			34.63 ***		

Note: Exposure: (1. Exposure; 0, non-exposure); course type: (1, Social Psychology; 2, Introduction to the Dialectics of Science); * *p* < 0.05, ** *p* < 0.01, *** *p* < 0.001.

## Data Availability

The original contributions presented in this study are included in the Supplementary Materials. Further inquiries can be directed to the corresponding author.

## Supplementary Materials

The following supporting information can be downloaded at https://www.mdpi.com/article/10.3390/bs15060731/s1. original data—pop culture.sav.
